# Cost-effectiveness of screening smokers and ex-smokers for lung cancer in the Netherlands in different age groups

**DOI:** 10.1007/s10198-021-01422-w

**Published:** 2022-01-05

**Authors:** Mohamed N. M. T. Al Khayat, Job F. H. Eijsink, Maarten J. Postma, Ewoudt M. W. van de Garde, Marinus van Hulst

**Affiliations:** 1 Department of Health Sciences, University of Groningen, University Medical Center Groningen, Groningen, The Netherlands; 2 Isala Hospital Zwolle, Zwolle, The Netherlands; 3 Department of Economics, Econometrics and Finance, Faculty of Economics and Business, University of Groningen, Groningen, The Netherlands; 4 Department of Clinical Pharmacy, St. Antonius Hospital, Utrecht, The Netherlands; 5 Department of Pharmacy, Unit of PharmacoTherapy, Epidemiology and Economics, University of Groningen, Groningen, The Netherlands; 6 Division of Pharmacoepidemiology and Clinical Pharmacology, Department of Pharmaceutical Sciences, Utrecht University, Utrecht, The Netherlands; 7 Department of Clinical Pharmacy, Martini Hospital Groningen, Groningen, The Netherlands

**Keywords:** Cost-effectiveness, Screening, Lung cancer, Smokers, Ex-smokers, I18, D04

## Abstract

**Objective:**

We aimed to assess the cost-effectiveness of screening smokers and ex-smokers for lung cancer in the Netherlands.

**Methods:**

A Markov model was used to evaluate the health effects and costs of lung cancer screening from the healthcare perspective. The effects and costs of ten screening scenarios with different start and stop ages of screening were examined across a lifetime horizon in a cohort of 100,000 smokers and ex- smokers 50 years and older.

**Results:**

The incremental cost-effectiveness ratios (ICERs) of screening smokers and ex-smokers aged 50–60 years, 50–70 years, and 50 years and older are below the cost-effectiveness threshold of € 20,000 per quality adjusted life year (QALY) gained. Screening 50–60-year-old smokers and ex-smokers was the most cost-effective scenario with an ICER of € 14,094 per QALY gained. However, screening smokers and ex-smokers 50 years and older yielded the highest QALYs and resulted in an ICER of € 16,594 per QALY, which is below the threshold of € 20,000 per QALY. All screening scenarios compared to no screening resulted in CERs between the € 14,000 and € 16,000 per QALY gained. The efficiency frontier showed that screening smokers and ex-smokers in the age groups 70 years and older, 60–70 years, 60 years and older are excluded by extended dominance by no screening, screening smokers and ex-smokers aged 50–60 years and 50–70 years.

**Conclusion:**

This study showed that lung cancer screening is cost-effective in the Netherlands.

**Supplementary Information:**

The online version contains supplementary material available at 10.1007/s10198-021-01422-w.

## Introduction

The burden of lung cancer in the world is high. According to the International Agency for Research on Cancer (IARC), the number of new cases in 2018 is more than 2 million (11.6% of total cancers) and the number of lung cancer deaths in 2018 worldwide is about 1.8 million (18.4% of total cancer mortality). Therefore, lung cancer is the most common cancer in the world with highest mortality rate [1]. The Dutch cancer registration estimated that lung cancer prevalence among men is 11.9% and 10.9% among women of total cancers in the Netherlands [2]. The mortality of lung cancer in the Netherlands is 72.2 for men (per 100,000 men) and 49.3 for women (per 100,000 women) in 2017 [3]. The majority (≈85%) of lung cancer patients are former or current smokers [4]. Lung cancer screening of smokers may be an option to reduce the burden of lung cancer. In the USA, lung cancer screening with low-dose computed tomography (LDCT) is already reimbursed for smokers and ex-smokers [5]. According to the WHO data of 2015, the prevalence of smoking in the Netherlands is higher than the USA (the Netherlands 26.2%, USA 19.5%) [6].

The NELSON trial in the Netherlands and Belgium showed that lung cancer screening reduced the lung cancer mortality by 26 percent (9–41%, 95% CI) among asymptomatic men at high risk for lung cancer [7]. The rate-ratio of dying among women from lung cancer was 0.73 at 10-year follow-up, indicating a larger reduction in lung cancer mortality than in men [8]. The reduction in mortality of lung cancer by implementing screening is due to the early detection thus treatment of lung cancer.

Results of the NELSON trial showed that implementing lung cancer screening will reduce the mortality due to lung cancer. To consider implementation of a lung cancer screening program among smokers and former smokers, the cost-effectiveness of such a program should be considered. The inclusion criteria of the screening program, follow-up, effectiveness of therapy, cost of therapy, prevalence after screening are important factors which affect the cost-effectiveness of such a screening program. The currently available immunotherapy treatment prolongs the survival of lung cancer patients. However, immunotherapy treatment is an expensive and not curative treatment. Surgery in early stage of lung cancer is a cheaper and curative treatment. Implementation of a lung cancer screening program for smokers and former smokers may therefore provide more access to curative treatments in early stage of lung cancer and hence prolonged survival.

The objective of this study is to assess the cost-effectiveness of implementing a lung cancer screening program in the Netherlands. The estimated cost-effectiveness may guide policy makers and guideline developers to decide whether lung cancer screening should be implemented and which groups are to be screened.

## Methods

### Model

**Fig. 1:**
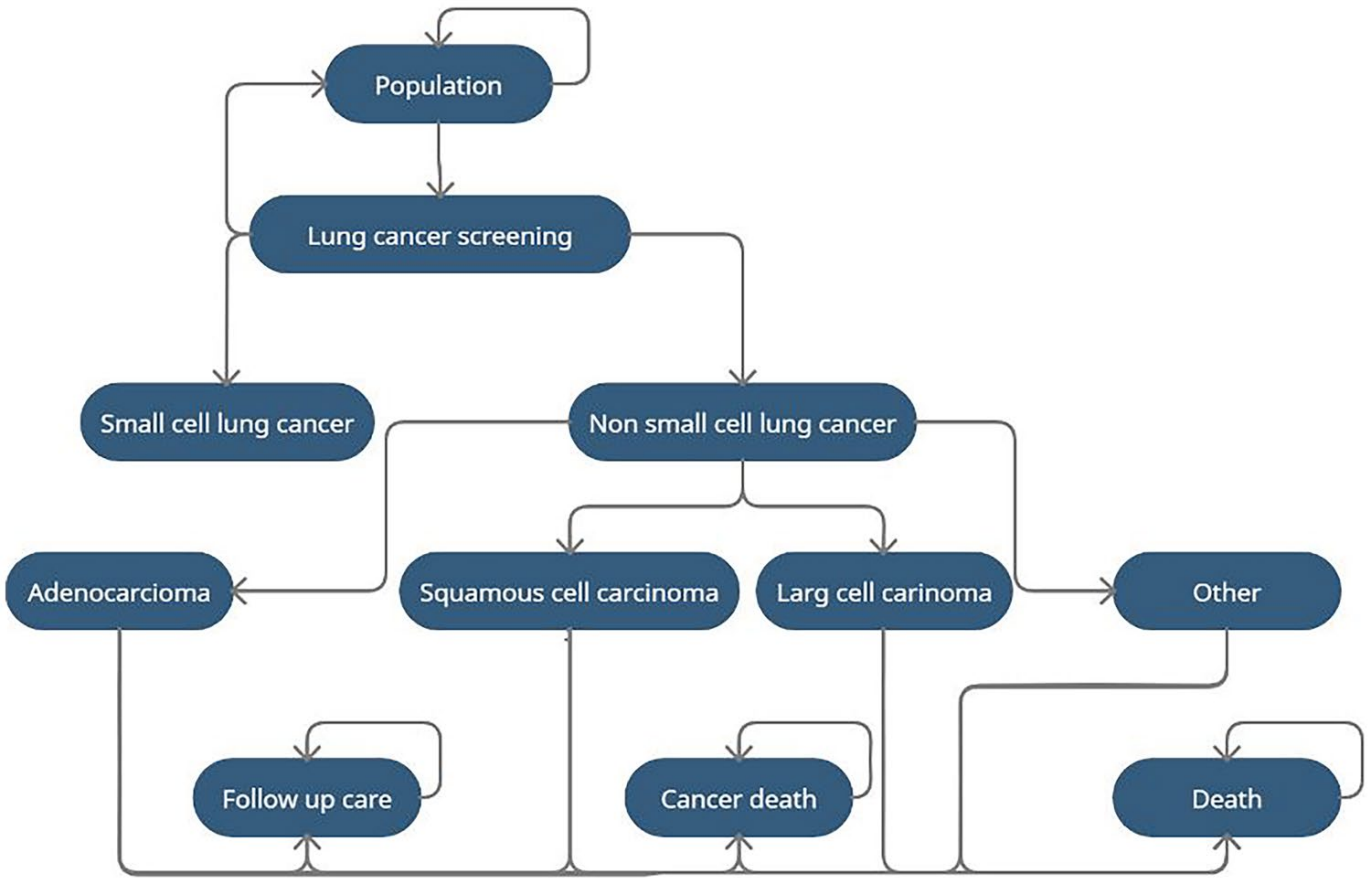
Markov model for natural history of lung cancer to evaluate the health and cost implications of screening

To estimate the cost-effectiveness of lung cancer screening in the Netherlands, we modelled the natural history of lung cancer combined with the potential effect of lung cancer screening in a Markov model. Notably, the Markov model presented in Fig. 1 was developed using R. We modelled a lifetime horizon using the WHO mortality rate in the Netherlands and we used an annual cycle to model the progression of lung cancer.

The probability to cure individuals is dependent on the histology and the stage of lung cancer. The model considers the probability of Small Cell Lung Cancer (SCLC) and Non-Small Cell Lung Cancer (NSCLC). NSCLC is divided into four histological types of lung cancer: adenocarcinoma, squamous cell carcinoma, large cell carcinoma and other NSCLC. In each type of lung cancer, the stages from IA to IV are defined. We used data from Integraal Kankercentrum Nederland (IKNL; Netherlands Comprehensive Cancer Organisation) to predict the prevalence in each stage of lung cancer for smokers and ex-smokers without screening. In case of screening, we used prevalence data from the NELSON trial. We assumed that individuals detected with screening at an early stage of lung cancer would be otherwise detected at one of the last stages of lung cancer. Moreover, we considered the probability of recurrence of cancer in the follow-up period. The transition-state probabilities used in this model are presented in Appendix S1.

### Population

To investigate the cost-effectiveness of lung cancer screening, we used age groups. Daily smoking behavior and percentage of smokers per age group is presented in Appendix S1 [9]. We included smokers or ex-smokers who smoked more than 30 years at least 15 cigarettes per day, with no more than 10 years since smoking cessation. These inclusion criteria are in line with the NELSON trial inclusion criteria. We used a fixed number of 10,000 people in every age group and used prevalence and age distribution to estimate the effectiveness of lung cancer screening.

The IKNL provided prevalence, mortality and 1-year lung cancer survival rates stratified by age, histology and stage. These data were used to estimate the prevalence of lung cancer in case of no screening, the distribution and survival according to lung cancer histology and stage. The NELSON trial data were used to estimate prevalence in case of screening. We used screen distribution of round 2 of NELSON trial with 1-year interval in line with our strategy of annual screening.

### Screening scenarios

We combined different characteristics of screening strategies such as start age of screening and stop age of screening. We chose start ages of 50, 60, 70 years and stop ages of 60, 70, life time. We used an annual screening interval in all combinations in line with United States Preventive Services Task Force (USPSTF) recommendations and currently available evidence [10, 11]. All scenarios were compared to the next least expensive screening strategy. Additionally, screening strategies were compared to no screening.

The screening program was assumed to use LDCT. Positive individuals will be tested by a PETscan to confirm the diagnosis and to avoid false positive results. Individuals with positive results of LDCT and PETscan will have biopsies to determine the histology of the tumor. Using three diagnostic tests LDCT, PETscan and biopsies, we assumed that false-positive results are nil. The false-negative results were assumed to be found in the next screening round. We assumed no lost to follow-up in the different screening scenarios.

### Costs

Costs were analysed from the Dutch healthcare perspective. The costs of activities building up the screening program were extracted from the Dutch database of the Healthcare Authority (Nederlandse zorgautoriteit) [12]. The input costs were calculated per histological type and stage of lung cancer. Input costs included costs of medications, surgery and palliative care. The input costs in the model are presented in Appendix S1. The cost of lung cancer drugs was estimated using the treatment duration and medication cost per unit. Treatment duration of chemotherapy was extracted from a recent study in the Santeon Hospital group to evaluate the treatment duration and intensity in lung cancer patients [13]. The Dutch database of the Healthcare Institute (Zorginstituut Nederland) was used to estimate medication costs per unit [13–15]. The treatment duration of immunotherapy was estimated from registration trials. A recent study of Cramer et al. confirmed that the treatment duration of immunotherapy in trials is comparable with the treatment duration of immunotherapy in a real world setting [15]. The costs of palliative care were calculated for the last six months of the terminal phase. These costs were extracted from the Dutch database of healthcare authority (Nederlandse zorgautoriteit) [12].

### Life expectancy and quality adjusted life years

We calculated the life expectancy of patients for different histological types and stages of lung cancer and adjusted for quality of life, using the reported utilities from Short Form Health Survey SF-36[16]. The reported utilities implied that utilities did not differ by age. Gareen et al. reported that there is no significant difference in health related quality of life or state anxiety at 1 or at 6 months after screening between participants receiving false-positive or true-negative results [17]. Therefore, we assumed that utilities did not differ by age of by receiving false-positive screening results.

We used the IKNL registered data to calculate the stage-specific mortality of lung cancer to estimate life expectancy after 1 year of diagnosis.

### Outcomes and cost-effectiveness

Using the Markov model, we assessed for each scenario the total discounted quality adjusted life-years (QALYs), total discounted cost, discounted QALYs gained, discounted incremental costs, ICER and the budget impact. The scenarios are sorted based on increasing total discounted costs. We used annual discounting rates of 4% on costs and 1.5% for QALYs according to Dutch guidelines [18]. The discounted QALYs gained and discounted incremental costs were calculated by comparing the total discounted costs and total discounted QALYs compared to the next least expensive scenario. ICERs were calculated as the total discounted incremental costs divided by the total discounted QALYs. In the Netherlands, there is no formal Dutch willingness-to-pay (WTP) threshold. Since screening programs can be considered a (secondary) preventive measure and screening generally faces most strict thresholds, we chose a conservative WTP threshold of €20,000 per QALY gained as reference point to determine which screening strategy is cost-effective [19].

### Sensitivity analyses

The impact of the transition-states, prevalence, costs and utilities on the ICER was analyzed by varying the parameters within their confidence interval or by 10% compared to used value. Monte Carlo simulations were used to estimate the uncertainty of ICER on the parameters used. We used 5000 simulations in the probabilistic whereby parameters were varied in the given ranges using a beta or gamma distribution. All varied parameters, distributions and range are given in the Appendix S1. The probabilistic analyses were further used to generate the cost-effectiveness acceptability frontier (CEAF).

## Results

**Fig. 2:**
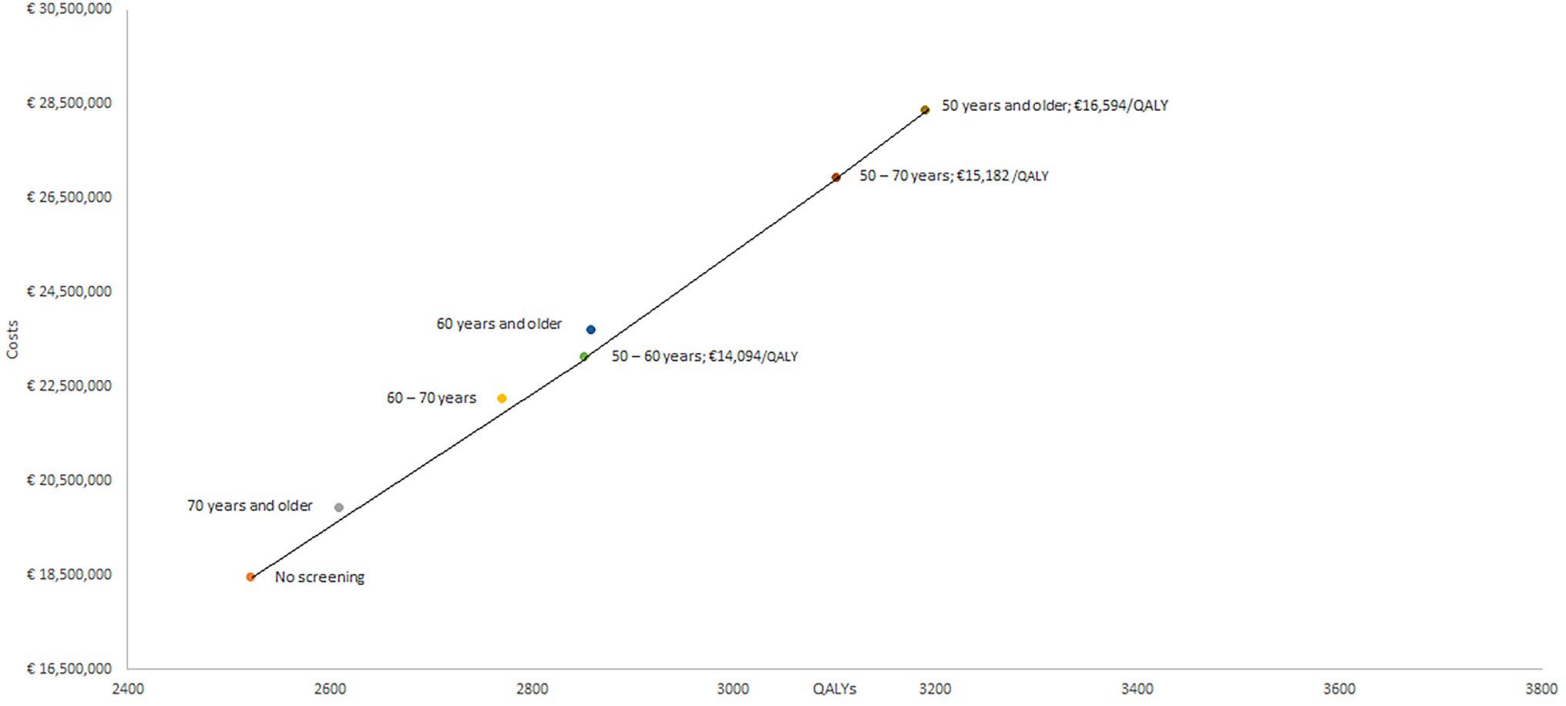
Cost-effectiveness efficiency frontier of lung cancer screening scenarios. QALY: quality adjusted life years; the incremental cost-effectiveness ratio (ICERs) are shown for each screening strategy. ICERs were calculated by comparing scenarios to the next least expensive screening strategy

The discounted costs and QALYs for each scenario versus no screening scenario are described in Table 1 and were used to plot the efficiency frontier in Fig. 2. All results were reported per 100,000 individuals with annual screening. The efficiency frontier in Fig. 2 shows that screening smokers and ex-smokers aged 70 years and older and 60–70 years are eliminated by extended dominance by no screening and screening smokers and ex-smokers aged 50–60 years. Moreover, screening smokers and ex-smokers aged 60 years and older is eliminated by extended dominance by screening smokers and ex-smokers aged 50–60 years and screening smokers and ex-smokers aged 50–70 years.

**Table 1:** Cost-effectiveness of lung cancer screening per 100,000 smokers or previous smokers

Screening scenario (age groups)	Costs	QALYs	ICERs	Costs versus no screening	QALYs gained versus no screening	CERs
No screening	€ 18,475,224	2520.9				
70 years and older	€ 19,931,407	2608.7	*	€ 1,456,183	87.8	€ 16,594
60–70 years	€ 22,264,907	2770.5	*	€ 3,789,683	249.6	€ 15,182
50–60 years	€ 23,143,766	2852.2	€ 14,094	€ 4,668,542	331.2	€ 14,094
60 years and older	€ 23,721,090	2858.3	*	€ 5,245,866	337.4	€ 15,549
50–70 years	€ 26,933,449	3101.8	€ 15,182	€ 8,458,225	580.9	€ 14,561
50 years and older	€ 28,389,632	3189.5	€ 16,594	€ 9,914,408	668.6	€ 14,828

*Extended dominated scenario; *QALY* Quality adjusted life years, *ICER* incremental cost-effectiveness ratio, *CER* cost-effectiveness ratio

All scenarios evaluated for lung cancer screening in smokers and ex-smokers were below the cost-effectiveness threshold of € 20,000 per QALY gained. Screening smokers and ex-smokers 70 years and older, 60–70 years of age, 60 years and olderwere excluded by extended dominance, please see Fig. 2. Notably, screening smokers and ex-smokers aged 50–60 years had the lowest ICER of € 14,094 per QALY gained incremental to no screening. ICERs between the €14,000 and €16,600 per QALY gained were found for all other evaluated screening strategies. Comparing screening scenarios to no screening is expressed in cost-effectiveness ratios (CERs). All screening scenarios compared to no screening resulted in CERs between the € 14,000 and € 16,000 per QALY gained. Notably, screening smokers and ex-smokers 50–60 years of age resulted in the lowest CER and screening smokers and ex-smokers 70 and older resulted in the highest CER.

Since all scenarios resulted in comparable ICERs and CERs, policy makers could consider the scenario with the highest QALYs gain and therefore the largest number of detected lung cancer patients. This implies that screening smokers and ex-smokers 50 years and older could be considered as a feasible screening scenario.

### Sensitivity analyses

**Fig. 3:**
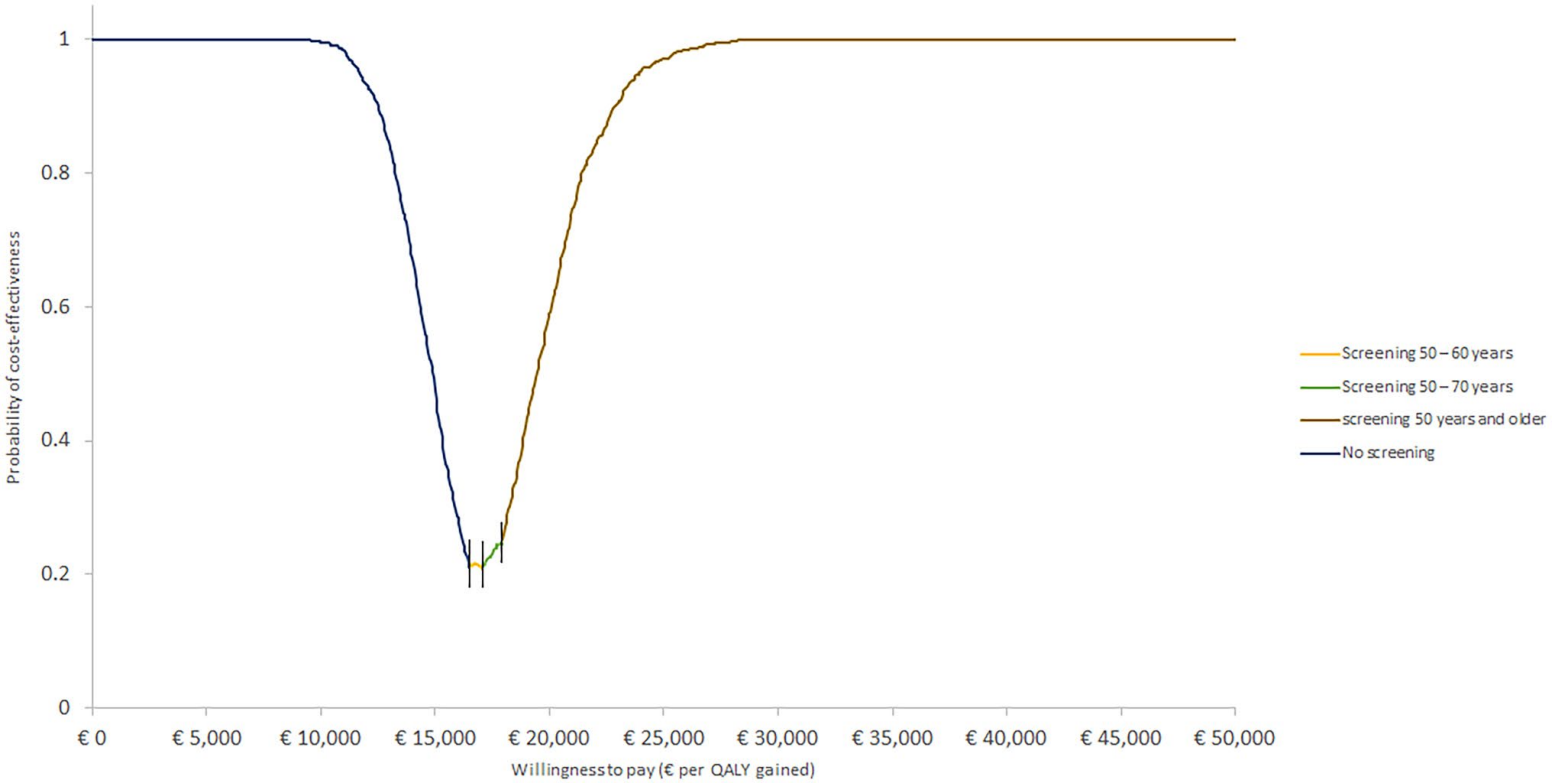
Cost-effectiveness acceptability frontier of lung cancer screening scenarios

The probabilistic sensitivity analysis was conducted for all scenarios. The resulting cost-effectiveness plane and cost-effectiveness acceptability curves (CEAC) are shown in Appendix S1. The CEAF presented in Fig. 3 shows that screening 50 years and older is the most cost-effective scenario using a willingness to pay (WTP) above the €17,950 per QALY. Screening 50–70 years scenario is the most cost-effective scenario using a WTP between €17,100 and €17,900 per QALY. For a WTP between €16,500 per QALY and €17,000 per QALY resulted in superior cost-effectiveness of screening 50–60 years old. Using a WTP below €16,500 per QALY resulted in the absence of screening as the most cost-effective scenario.

## Discussion

In this study, we showed that lung cancer screening is cost-effective in a population of smokers and ex-smokers. Screening of almost all age groups of smokers and ex-smokers resulted in cost-effectiveness ratios under the €20,000 per QALY gained threshold. This implies that lung cancer screening of smokers and ex-smokers is cost-effective in The Netherlands.

Our results showed, obviously, that screening program costs and QALYs increased when the screened population enlarged. Screening smokers and ex-smokers of 50 years and older resulted in the highest costs and QALYs while screening smokers and ex-smokers 70 and older resulted in the lowest costs and QALYs. Notably, screening start and stop age has significant impact on the cost-effectiveness of screening scenarios. Increasing the start age reduced the cost and benefits of lung cancer screening, while starting screening at younger age increased the cost and benefits of lung cancer screening. The efficiency frontier showed that screening 70 years and older, 60–70 years of age and 60 years and older smokers and ex-smokers is eliminated by extended dominance. These dominated scenarios are considered cost-ineffective since the alternative scenarios are more cost-effective. The most cost-effective scenario is screening 50–60-year-old smokers and ex-smokers, which resulted in an ICER of € 14,094 per QALY gained compared to no screening.

The cost-effectiveness acceptability frontier confirms that screening 50–60-year-old smokers and ex-smokers is the most cost-effective scenario using WTP between €16,500 per QALY and €17,000 per QALY. Using a higher WTP resulted obviously in cost-effectiveness of larger populations starting by screening 50–70-year-old smokers and ex-smokers followed by screening 50 years and older screening scenario.

A cost-effectiveness study in Germany yielded similar results of screening high-risk populations for lung cancer. Hofer et al. evaluated the cost-effectiveness of screening heavy current or former smokers (≥ 20 cigarettes per day) aged between 55 and 75 years compared no screening resulted in ICER € 30,291 per QALY gained for lung cancer screening [20]. Hofer et al. did not include immunotherapy in the analysis, which may explain the higher ICER in that study compared to our results. Moreover, Hofer et al. chose a time horizon of 15 years instead of life time horizon. Hofer et al. reported that choosing a longer time horizon decreased the ICER. A cost-effectiveness study in Canada evaluated screening different age cohorts using NELSON trial scenarios. [21] This study resulted in an ICER of $48,000 per life years gained by screening smokers annually from 55 to 80 years. This study resulted in higher ICER because Ten Haaf et al. did not include the immunotherapy as a treatment option. Moreover, the lower healthcare costs in the Netherlands might led to a lower ICER in our study. The cost-effectiveness study of lung cancer screening in Switzerland by Tomonaga et al. evaluated the different NELSON trial scenarios and included the immunotherapy [22]. Tomonaga et al. evaluation resulted in ICERs between € 30,000 and € 40,000 per life years gained compared to no screening. This study also resulted in higher ICERs than our results because of the high healthcare cost in Switzerland compared to the Netherlands.

Our study has some important limitations. First, we did not include the adherence rate in our analysis. Therefore, our results may overestimate the effectiveness of screening programs. Including a low adherence rate would result in a higher cost-effectiveness ratio. Second, we did not include the side effects of screening in our model. Annual screening with LDCT could be a potential harm because of radiation exposure. However, a recent study by Bach et al. concluded that the potential benefit of lung cancer screening is higher that the potential harm of radiation exposure. [23] Third, we assumed the same surgery costs for all types of surgical interventions. However, we used the highest surgical costs and applied it to all surgical interventions. This might overestimate the ICER. Using lower surgical costs would result in lower ICER. Fourth, we did not include lead-time bias in our analyses. However, the effect of lead-time bias is minimized using Real world survival data from IKNL [24]. We applied real world survival data by age, stage and pathology to our screening and no screening population. This approach could underestimate the survival of patients found by screening and no screening in the same stage. This underestimation might balance the overestimation of possible lead-time bias caused by stage shift. Moreover, we did not include overdiagnosis in our model. Overdiagnosis may lead to unnecessary follow-up procedure and may cause anxiety. Therefore, our results could overestimate the effects of screening resulting in a lower ICER. Including these side-effects of screening could result in higher cost-effectiveness ratios.

## Conclusion

Our study indicated that lung cancer screening program for smokers and ex-smokers is cost-effective in the Netherlands. Our results may support policy makers and guideline developers who face the decision whether lung cancer screening should be implemented for smokers and ex-smokers.

## Supplementary Information

Below is the link to the electronic supplementary material.

Supplementary file1 (DOCX 343 KB)

## Data Availability

All authors approved that all data and materials support their published claims and comply with field standards. Data are available on reasonable request.

## Code availability

Code is available on reasonable request.

## References

[1] IARC—International Agency For Research On Cancer (2019). Available from: https://www.iarc.fr/

[2] Nederlandse Kankerregistratie (2019). Available from: https://www.cijfersoverkanker.nl/

[3] CBS (2019) Available from: https://www.volksgezondheidenzorg.info/onderwerp/longkanker/cijfers-context/sterfte-en-overleving#node-sterfte-longkanker-naar-leeftijd-en-geslacht

[4] Rahal, Z., El Nemr, S., Sinjab, A., Chami, H., Tfayli, A., Kadara, H.: Smoking and lung cancer: a geo-regional perspective. Front Oncol. 7:194 (2017). Available from: http://www.ncbi.nlm.nih.gov/pubmed/28920053 10.3389/fonc.2017.00194 PMC5585135 28920053

[5] Decision Memo for Screening for Lung Cancer with Low Dose Computed Tomography (LDCT) (CAG-00439N). 2015 Available from: Decision Memo for Screening for Lung Cancer with Low Dose Computed Tomography (LDCT) (CAG-00439N) (2019)

[6] WHO | World Health Organization (2019). Available from: http://gamapserver.who.int/gho/interactive_charts/tobacco/use/atlas.html

[7] Rivero, L., Bunn, B.: NELSON study shows CT screening for nodule volume management reduces lung cancer mortality by 26 percent in Men (2019). Available from: www.iaslc.org

[8] De, Koning, H., Van Der, Aalst, C., Ten, Haaf, K., Oudkerk, M.: PL02.05 Effects of volume ct lung cancer screening: mortality results of the NELSON randomised-controlled population based trial. J. Thorac. Oncol. 13(10):S185 (2018). Available from: https://www.sciencedirect.com/science/article/pii/S1556086418309705

[9] Roken onder volwassenen | RIVM (2021). Available from: https://www.rivm.nl/leefstijlmonitor/roken-onder-volwassenen

[10] Krist AH, Davidson KW, Mangione CM, Barry MJ, Cabana M, Caughey AB (2021). Screening for lung cancer: US preventive services task force recommendation statement. JAMA-J Am Med Assoc..

[11] Heuvelmans MA, Oudkerk M (2018). Appropriate screening intervals in low-dose CT lung cancer screening. Transl Lung Cancer Res..

[12] NZa zorgproductapplicatie (2019). Available from: https://zorgproducten.nza.nl/Home.aspx

[13] Cramer-Van Der Welle CM, Peters BJM, Schramel FMNH, Klungel OH, Groen HJM, Van De Garde EMW (2018). Systematic evaluation of the efficacy-effectiveness gap of systemic treatments in metastatic nonsmall cell lung cancer. Eur Respir J..

[14] Welkom bij Medicijnkosten (2019). Available from: https://www.medicijnkosten.nl/

[15] Cramer-van der Welle CM, Verschueren MV, Tonn M, Peters BJM, Schramel FMNH, Klungel OH (2021). Real-world outcomes versus clinical trial results of immunotherapy in stage IV non-small cell lung cancer (NSCLC) in the Netherlands. Sci Rep.

[16] Black, W.C., Gareen, I.F., Soneji, S.S., Sicks, J.D., Keeler, E.B., Aberle, D.R., et al.: Cost-effectiveness of CT screening in the national lung screening trial. N Engl. J. Med. 371(19):1793–802 (2014). Available from: http://www.nejm.org/doi/10.1056/NEJMoa1312547 10.1056/NEJMoa1312547 PMC4335305 25372087

[17] Gareen IF, Duan F, Greco EM, Snyder BS, Boiselle PM, Park ER (2014). Impact of lung cancer screening results on participant health-related quality of life and state anxiety in the National Lung Screening Trial. Cancer.

[18] Richtlijn voor het uitvoeren van economische evaluaties in de gezondheidszorg | Publicatie | Zorginstituut Nederland (2019). Available from: https://www.zorginstituutnederland.nl/over-ons/publicaties/publicatie/2016/02/29/richtlijn-voor-het-uitvoeren-van-economische-evaluaties-in-de-gezondheidszorg

[19] Kosteneffectiviteit in de praktijk | Rapport | Zorginstituut Nederland (2019). Available from: https://www.zorginstituutnederland.nl/publicaties/rapport/2015/06/26/kosteneffectiviteit-in-de-praktijk

[20] Hofer, F., Kauczor, H.U., Stargardt, T.: Cost-utility analysis of a potential lung cancer screening program for a high-risk population in Germany: A modelling approach. Lung Cancer. 124:189–98 (2018). Available from: https://pubmed.ncbi.nlm.nih.gov/30268459/10.1016/j.lungcan.2018.07.036 30268459

[21] ten Haaf, K., Tammemägi, M,C., Bondy, S.J., van der Aalst, C.M., Gu, S., McGregor, S.E., et al.: Performance and cost-effectiveness of computed tomography lung cancer screening scenarios in a population-based setting: a microsimulation modeling analysis in Ontario, Canada. PLoS Med. 14(2) (2017). Available from: https://pubmed.ncbi.nlm.nih.gov/28170394/10.1371/journal.pmed.1002225 PMC5295664 28170394

[22] Tomonaga, Y., ten Haaf, K., Frauenfelder, T., Kohler, M., Kouyos, R.D., Shilaih, M., et al.: Cost-effectiveness of low-dose CT screening for lung cancer in a European country with high prevalence of smoking—a modelling study. Lung Cancer. 121:61–9 (2018). Available from: https://pubmed.ncbi.nlm.nih.gov/29858029/10.1016/j.lungcan.2018.05.008 29858029

[23] Bach, P.B., Mirkin, J.N., Oliver, T.K., Azzoli, C.G., Berry, D.A., Brawley, O.W., et al.: Benefits and harms of CT screening for lung cancer: a systematic review. JAMA-J. Am. Med. Assoc. 307:2418–29 (2012). Available from: https://jamanetwork.com/journals/jama/fullarticle/1163892 10.1001/jama.2012.5521 PMC3709596 22610500

[24] Yang, S.C., Wang, J, Der., Wang, S.Y.: Considering lead-time bias in evaluating the effectiveness of lung cancer screening with real-world data. Sci Rep. 11(1):1–10 (2021). Available from: https://www.nature.com/articles/s41598-021-91852-6 10.1038/s41598-021-91852-6 PMC8190256 34108586

