# Supplementary material for: Cost-effectiveness of screening smokers and ex-smokers for lung cancer in the Netherlands in different age groups
Source: Eur J Health Econ. 2022 Jan 5;23(7):1221–7. doi: 10.1007/s10198-021-01422-w (PMC9395469; doi:10.1007/s10198-021-01422-w)
Supplement: Supplementary file 1 — Supplementary file1 (DOCX 343 KB) [file 10198_2021_1422_MOESM1_ESM.docx]

Table 1 Transition probabilities of lung cancer

| **From** | **To** | **Value** | **Reference** |
| --- | --- | --- | --- |
| Lung carcinoma screening | Non-small cell lung | 0.85 | [1] |
|  | Small cell lung | 0.15 | [1] |
| Non-small cell lung | Adenocarcinoma | 0.4 | [2] |
|  | Squamous cell carcinoma | 0.3 | [2] |
|  | Large cell carcinoma | 0.15 | [2] |
|  | Other | 0.15 | [2] |
| Screening: Small cell lung/adenocarcinoma/Squamous cell carcinoma/Large cell carcinoma/Other | Stage 1 | 0.759 | [3] |
|  | Stage 2 | 0.069 | [3] |
|  | Stage 3 | 0.138 | [3] |
|  | Stage 4 | 0.034 | [3] |
| No screening: Small cell lung/adenocarcinoma/Squamous cell carcinoma/Large cell carcinoma/Other | Stage 1 | 0.26 | [4] |
|  | Stage 2 | 0.11 | [4] |
|  | Stage 3 | 0.26 | [4] |
|  | Stage 4 | 0.37 | [4] |

Table 2 Age cohorts with corresponding prevalence of daily smokers and percentage of smoking population in the Netherland. This table is informative, the data is not used in the model.

| **Cohort** | **Prevalence daily smokers** | **Percentage of smoking population** |
| --- | --- | --- |
| 40-49 years | 17.5% - 20.3% | 32% |
| 50-59 years | 18.5%-19.2% | 32% |
| 60-69 years | 12.9%-18.5% | 26% |
| 70+ years | 2.5%-9.2% | 10% |

Table 3 Costs parameters

| **Type of costs** | **Unit costs in €** | **Reference** |
| --- | --- | --- |
| Screening programme |  |  |
| 1. LDCT | € 175 | [5] |
| 1. PET scan | € 1,109 | [5] |
| 1. Biopsy | € 638 | [5] |
| Treatment NSCLC |  |  |
| 1. Stage 1 | € 15,282 | [6–9] |
| 1. Stage 2 | € 21,560 | [6–9] |
| 1. Stage 3 | € 22,240 | [6–9] |
| 1. Stage 4 | € 23,070 | [6–9] |
| Treatment SCLC |  |  |
| 1. Stage 1 | € 15,282 | [6–8] |
| 1. Stage 2 | € 21,560 | [6–8] |
| 1. Stage 3 | € 22,240 | [6–8] |
| 1. Stage 4 | € 7,800 | [6–8] |
| Palliative care | € 6,300 | [5,8] |

LDCT: low-dose computed tomography; NSCLC: Non-small cell lung carcinoma; SCLC: Small cell lung carcinoma

Table 4 Costs of different treatment options of lung cancer

| **Non-small cell lung carcinoma** | | | | | |
| --- | --- | --- | --- | --- | --- |
| **Stage** | **Treatment** | **Percentage** | **Costs** | **Total costs** | **Reference** |
| Stage 1 | Surgery | 90% | € 14,400 | € 15,283 | [6–9] |
|  | surgery + chemotherapy | 5% | € 20,450 |  |  |
|  | surgery + chemotherapy + radiotherapy | 5% | € 26,000 |  |  |
| Stage 2 | surgery + chemotherapy | 80% | € 20,450 | € 21,560 | [6–9] |
|  | surgery + chemotherapy + radiotherapy | 20% | € 26,000 |  |  |
| Stage 3 | surgery + chemotherapy + radiotherapy | 20% | € 26,000 | € 22,240 | [6–9] |
|  | chemotherapy + radiotherapy | 80% | € 21,300 |  |  |
| Stage 4 | chemotherapy + radiotherapy | 10% | € 21,300 | € 23,070 | [5–9] |
|  | chemotherapy + radiotherapy + immunotherapy | 30% | € 57,200 |  |  |
|  | palliative care | 60% | € 6,300 |  |  |
| **Small cell lung carcinoma** | | | | | |
| **Stage** | **Treatment** | **Percentage** | **Costs** | **Total costs** | **Reference** |
| Stage 1 | Surgery | 90% | € 14,400 | € 15,283 | [6–8]  [6–8]  [6–8] |
|  | surgery + chemotherapy | 5% | € 20,450 |  |  |
|  | surgery + chemotherapy + radiotherapy | 5% | € 26,000 |  |  |
| Stage 2 | surgery + chemotherapy | 80% | € 20,450 | € 21,560 | [6–8] |
|  | surgery + chemotherapy + radiotherapy | 20% | € 26,000 |  |  |
| Stage 3 | surgery + chemotherapy + radiotherapy | 20% | € 26,000 | € 22,240 | [6–8] |
|  | chemotherapy + radiotherapy | 80% | € 21,300 |  |  |
| Stage 4 | chemotherapy + radiotherapy | 10% | € 21,300 | € 7,800 | [5–8] |
|  | palliative care | 90% | € 6,300 |  |  |

Table 5 Sensitivity analysis parameters

| Parameter | Value | Low | High | Distribution | Reference |
| --- | --- | --- | --- | --- | --- |
| Prevalence stage 1 screening | 0.759 | 0.6831 | 0.8349 | Beta | [3] |
| Prevalence stage 2 screening | 0.069 | 0.0621 | 0.0759 | Beta | [3] |
| Prevalence stage 3 screening | 0.138 | 0.1242 | 0.1518 | Beta | [3] |
| Prevalence stage 4 screening | 0.034 | 0.0306 | 0.0374 | Beta | [3] |
| Prevalence stage 1 no screening | 0.25 | 0.225 | 0.275 | Beta | [4] |
| Prevalence stage 2 no screening | 0.11 | 0.099 | 0.121 | Beta | [4] |
| Prevalence stage 3 no screening | 0.27 | 0.243 | 0.297 | Beta | [4] |
| Prevalence stage 4 no screening | 0.37 | 0.333 | 0.407 | Beta | [4] |
| Recurrence | 0.26 | 0.234 | 0.286 | Beta | [12] |
| Adenocarcinoma stage 1 cancer death by 40 years old | 0.050 | 0.007 | 0.305 | Beta | [4] |
| Adenocarcinoma stage 3 cancer death by 40 years old | 0.131 | 0.051 | 0.313 | Beta | [4] |
| Adenocarcinoma stage 4 cancer death by 40 years old | 0.548 | 0.454 | 0.648 | Beta | [4] |
| Squamous cell carcinoma stage 3 cancer death by 40 years old | 0.143 | 0.021 | 0.666 | Beta | [4] |
| Squamous cell carcinoma stage 4 cancer death by 40 years old | 0.632 | 0.352 | 0.900 | Beta | [4] |
| Large cell carcinoma stage 1 cancer death by 40 years old | 0.400 | 0.068 | 0.975 | Beta | [4] |
| Large cell carcinoma stage 3 cancer death by 40 years old | 0.118 | 0.017 | 0.589 | Beta | [4] |
| Large cell carcinoma stage 4 cancer death by 40 years old | 0.600 | 0.372 | 0.835 | Beta | [4] |
| Other cell carcinoma stage 4 cancer death by 40 years old | 0.500 | 0.155 | 0.942 | Beta | [4] |
| Small cell lung carcinoma stage 4 cancer death by 40 years old | 0.600 | 0.372 | 0.835 | Beta | [4] |
| Adenocarcinoma stage 1 cancer death by 50 years old | 0.046 | 0.019 | 0.106 | Beta | [4] |
| Adenocarcinoma stage 2 cancer death by 50 years old | 0.107 | 0.041 | 0.260 | Beta | [4] |
| Adenocarcinoma stage 3 cancer death by 50 years old | 0.222 | 0.152 | 0.318 | Beta | [4] |
| Adenocarcinoma stage 4 cancer death by 50 years old | 0.603 | 0.556 | 0.650 | Beta | [4] |
| Squamous cell carcinoma stage 1 cancer death by 50 years old | 0.095 | 0.025 | 0.330 | Beta | [4] |
| Squamous cell carcinoma stage 2 cancer death by 50 years old | 0.069 | 0.010 | 0.398 | Beta | [4] |
| Squamous cell carcinoma stage 3 cancer death by 50 years old | 0.381 | 0.274 | 0.512 | Beta | [4] |
| Squamous cell carcinoma stage 4 cancer death by 50 years old | 0.785 | 0.668 | 0.883 | Beta | [4] |
| Large cell carcinoma stage 1 cancer death by 50 years old | 0.111 | 0.016 | 0.567 | Beta | [4] |
| Large cell carcinoma stage 3 cancer death by 50 years old | 0.514 | 0.366 | 0.680 | Beta | [4] |
| Large cell carcinoma stage 4 cancer death by 50 years old | 0.689 | 0.599 | 0.776 | Beta | [4] |
| Other cell carcinoma stage 3 cancer death by 50 years old | 0.381 | 0.163 | 0.725 | Beta | [4] |
| Other cell carcinoma stage 4 cancer death by 50 years old | 0.842 | 0.651 | 0.961 | Beta | [4] |
| Small cell lung carcinoma stage 2 cancer death by 50 years old | 0.500 | 0.155 | 0.942 | Beta | [4] |
| Small cell lung carcinoma stage 3 cancer death by 50 years old | 0.227 | 0.129 | 0.381 | Beta | [4] |
| Small cell lung carcinoma stage 4 cancer death by 50 years old | 0.664 | 0.577 | 0.749 | Beta | [4] |
| Adenocarcinoma stage 1 cancer death by 60 years old | 0.063 | 0.038 | 0.104 | Beta | [4] |
| Adenocarcinoma stage 2 cancer death by 60 years old | 0.036 | 0.012 | 0.109 | Beta | [4] |
| Adenocarcinoma stage 3 cancer death by 60 years old | 0.297 | 0.239 | 0.367 | Beta | [4] |
| Adenocarcinoma stage 4 cancer death by 60 years old | 0.626 | 0.594 | 0.658 | Beta | [4] |
| Squamous cell carcinoma stage 1 cancer death by 60 years old | 0.088 | 0.045 | 0.169 | Beta | [4] |
| Squamous cell carcinoma stage 2 cancer death by 60 years old | 0.180 | 0.104 | 0.302 | Beta | [4] |
| Squamous cell carcinoma stage 3 cancer death by 60 years old | 0.359 | 0.292 | 0.437 | Beta | [4] |
| Squamous cell carcinoma stage 4 cancer death by 60 years old | 0.646 | 0.576 | 0.715 | Beta | [4] |
| Large cell carcinoma stage 1 cancer death by 60 years old | 0.095 | 0.025 | 0.330 | Beta | [4] |
| Large cell carcinoma stage 2 cancer death by 60 years old | 0.080 | 0.012 | 0.447 | Beta | [4] |
| Large cell carcinoma stage 3 cancer death by 60 years old | 0.389 | 0.289 | 0.509 | Beta | [4] |
| Large cell carcinoma stage 4 cancer death by 60 years old | 0.736 | 0.676 | 0.793 | Beta | [4] |
| Other cell carcinoma stage 1 cancer death by 60 years old | 0.087 | 0.013 | 0.476 | Beta | [4] |
| Other cell carcinoma stage 2 cancer death by 60 years old | 0.308 | 0.087 | 0.773 | Beta | [4] |
| Other cell carcinoma stage 3 cancer death by 60 years old | 0.182 | 0.049 | 0.553 | Beta | [4] |
| Other cell carcinoma stage 4 cancer death by 60 years old | 0.667 | 0.507 | 0.818 | Beta | [4] |
| Small cell lung carcinoma stage 2 cancer death by 60 years old | 0.143 | 0.021 | 0.666 | Beta | [4] |
| Small cell lung carcinoma stage 3 cancer death by 60 years old | 0.361 | 0.272 | 0.468 | Beta | [4] |
| Small cell lung carcinoma stage 4 cancer death by 60 years old | 0.649 | 0.597 | 0.701 | Beta | [4] |
| Adenocarcinoma stage 1 cancer death by 70 years old | 0.088 | 0.060 | 0.129 | Beta | [4] |
| Adenocarcinoma stage 2 cancer death by 70 years old | 0.146 | 0.090 | 0.230 | Beta | [4] |
| Adenocarcinoma stage 3 cancer death by 70 years old | 0.366 | 0.310 | 0.428 | Beta | [4] |
| Adenocarcinoma stage 4 cancer death by 70 years old | 0.718 | 0.692 | 0.744 | Beta | [4] |
| Squamous cell carcinoma stage 1 cancer death by 70 years old | 0.188 | 0.134 | 0.259 | Beta | [4] |
| Squamous cell carcinoma stage 2 cancer death by 70 years old | 0.245 | 0.181 | 0.327 | Beta | [4] |
| Squamous cell carcinoma stage 3 cancer death by 70 years old | 0.489 | 0.437 | 0.542 | Beta | [4] |
| Squamous cell carcinoma stage 4 cancer death by 70 years old | 0.766 | 0.721 | 0.809 | Beta | [4] |
| Large cell carcinoma stage 1 cancer death by 70 years old | 0.240 | 0.133 | 0.411 | Beta | [4] |
| Large cell carcinoma stage 2 cancer death by 70 years old | 0.217 | 0.097 | 0.446 | Beta | [4] |
| Large cell carcinoma stage 3 cancer death by 70 years old | 0.534 | 0.444 | 0.629 | Beta | [4] |
| Large cell carcinoma stage 4 cancer death by 70 years old | 0.797 | 0.751 | 0.840 | Beta | [4] |
| Other cell carcinoma stage 1 cancer death by 70 years old | 0.216 | 0.087 | 0.478 | Beta | [4] |
| Other cell carcinoma stage 2 cancer death by 70 years old | 0.333 | 0.122 | 0.718 | Beta | [4] |
| Other cell carcinoma stage 3 cancer death by 70 years old | 0.444 | 0.269 | 0.668 | Beta | [4] |
| Other cell carcinoma stage 4 cancer death by 70 years old | 0.803 | 0.705 | 0.884 | Beta | [4] |
| Small cell lung carcinoma stage 1 cancer death by 70 years old | 0.308 | 0.128 | 0.627 | Beta | [4] |
| Small cell lung carcinoma stage 2 cancer death by 70 years old | 0.323 | 0.149 | 0.610 | Beta | [4] |
| Small cell lung carcinoma stage 3 cancer death by 70 years old | 0.547 | 0.463 | 0.635 | Beta | [4] |
| Small cell lung carcinoma stage 4 cancer death by 70 years old | 0.776 | 0.735 | 0.814 | Beta | [4] |
| Cost lung cancer stage 1 | € 15,282 | € 13,754 | € 16,810 | Gamma | [6,7,9,10] |
| Cost lung cancer stage 2 | € 21,560 | € 19,404 | € 23,716 | Gamma | [6,7,9,10] |
| Cost lung cancer stage 3 | € 22,250 | € 20,025 | € 24,475 | Gamma | [6,7,9,10] |
| Cost lung cancer stage 4 | € 127,252 | € 114,527 | € 139,977 | Gamma | [6,7,9-11] |
| CT screening | € 175 | € 158 | € 193 | Gamma | [5] |
| PET scan | € 1,109 | € 998 | € 1,220 | Gamma | [5] |
| Biopsy | € 638 | € 574 | € 702 | Gamma | [5] |
| Utility lung cancer stage 1 | 0.72 | 0.648 | 0.792 | Beta | [13] |
| Utility lung cancer stage 2 | 0.67 | 0.603 | 0.737 | Beta | [13] |
| Utility lung cancer stage 3 | 0.645 | 0.5805 | 0.7095 | Beta | [13] |
| Utility lung cancer stage 4 | 0.62 | 0.558 | 0.682 | Beta | [13] |
| Utility follow up lung cancer | 0.8 | 0.72 | 0.88 | Beta | Estimated |


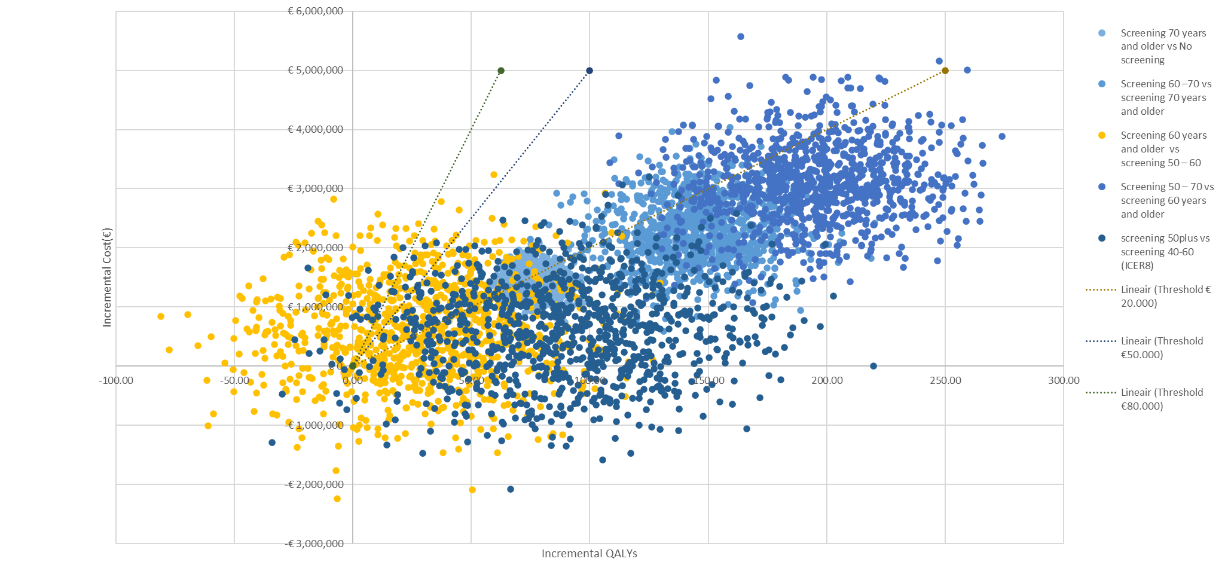


Figure 1 Cost-effectiveness plane of lung cancer screening programs. QALYs = quality adjusted life years. Each bullet demonstrates the costs and QALYs of a particular screening strategy. The dashed lines represent the different thresholds.


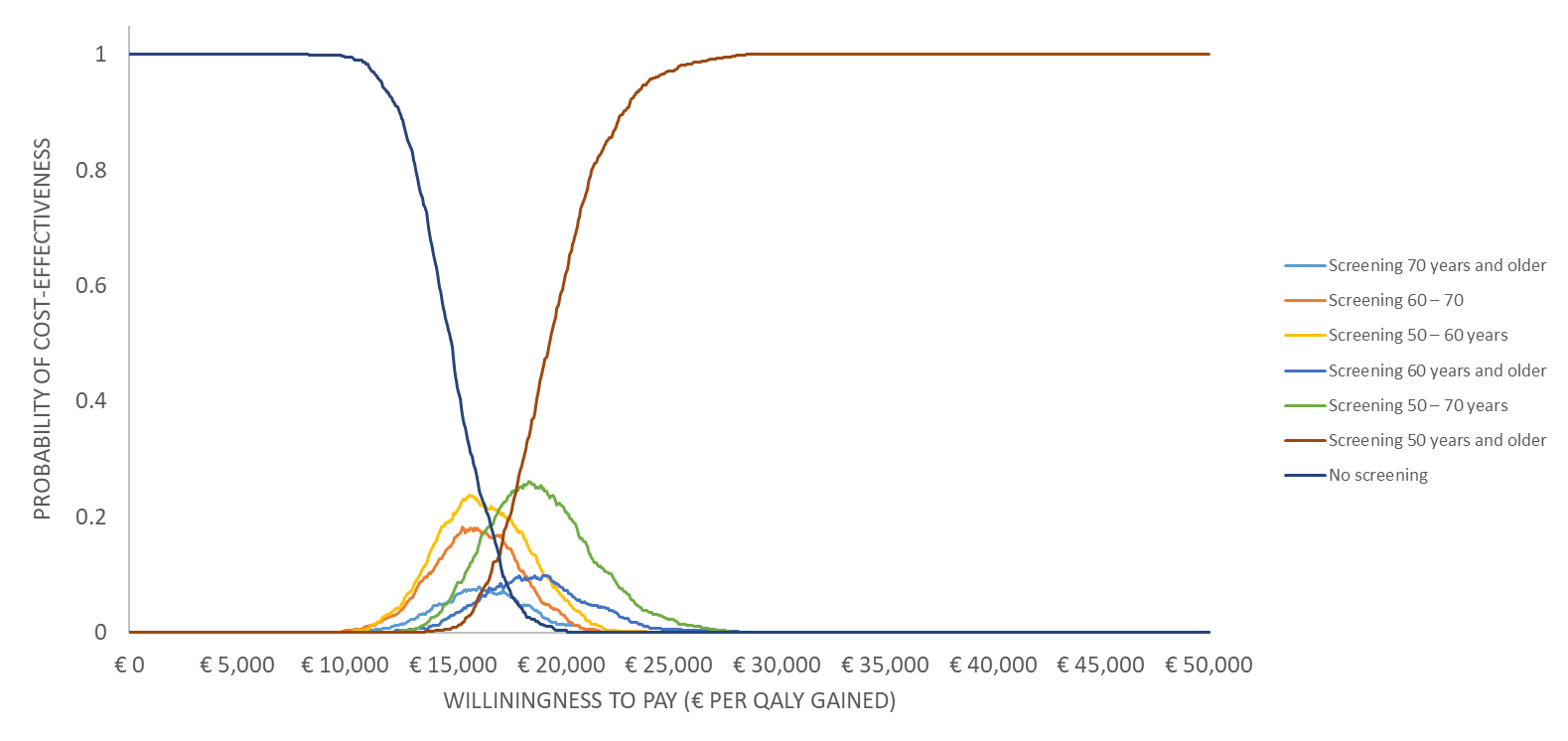


Figure 2 Cost-effectiveness acceptability curve of lung cancer screening programs

References

1. Molina JR, Yang P, Cassivi SD, Schild SE, Adjei AA. Non-small cell lung cancer: Epidemiology, risk factors, treatment, and survivorship. In: Mayo Clinic Proceedings. Elsevier Ltd; 2008. p. 584–94.

2. What Is Lung Cancer? | Types of Lung Cancer [Internet]. [cited 2019 Dec 11]. Available from: https://www.cancer.org/content/cancer/en/cancer/lung-cancer/about/what-is.html

3. Yousaf-Khan U, Van Der Aalst C, De Jong PA, Heuvelmans M, Scholten E, Lammers JW, et al. Final screening round of the NELSON lung cancer screening trial: The effect of a 2.5-year screening interval. Thorax. 2017 Jan 1;72(1):48–56.

4. Home - IKNL [Internet]. [cited 2021 Feb 5]. Available from: https://iknl.nl/

5. NZa zorgproductapplicatie [Internet]. [cited 2019 Dec 11]. Available from: https://zorgproducten.nza.nl/Home.aspx

6. Welkom bij Medicijnkosten [Internet]. [cited 2019 Dec 11]. Available from: https://www.medicijnkosten.nl/

7. Schwarzkopf L, Wacker M, Holle R, Leidl R, Günster C, Adler JB, et al. Cost-components of lung cancer care within the first three years after initial diagnosis in context of different treatment regimens. Lung Cancer. 2015 Nov 1;90(2):274–80.

8. Hofer F, Kauczor HU, Stargardt T. Cost-utility analysis of a potential lung cancer screening program for a high-risk population in Germany: A modelling approach. Lung Cancer [Internet]. 2018 Oct 1 [cited 2021 Nov 12];124:189–98. Available from: https://pubmed.ncbi.nlm.nih.gov/30268459/

9. Cramer-van der Welle CM, Peters BJM, Deenen MJ, Schramel FMNH, van de Garde EMW. Trends in Drug Costs and Overall Survival in Patients with Metastatic Non-small Cell Lung Cancer in The Netherlands Diagnosed from 2008 Through 2014. PharmacoEconomics - open [Internet]. 2021 Mar 1 [cited 2021 Dec 3];5(1):121–7. Available from: https://pubmed.ncbi.nlm.nih.gov/32533521/

10. Cramer-Van Der Welle CM, Peters BJM, Schramel FMNH, Klungel OH, Groen HJM, Van De Garde EMW. Systematic evaluation of the efficacy-effectiveness gap of systemic treatments in metastatic nonsmall cell lung cancer. Eur Respir J. 2018;52(6).

11. Lee SE, Kim YJ, Sung M, Lee MS, Han J, Kim HK, et al. Association with PD-L1 expression and clinicopathological features in 1000 lung cancers: A large single-institution study of surgically resected lung cancers with a high prevalence of EGFR mutation. Int J Mol Sci [Internet]. 2019 Oct 1 [cited 2021 Jan 15];20(19). Available from: /pmc/articles/PMC6801455/?report=abstract

12. Maurizi G, D’Andrilli A, Ciccone AM, Ibrahim M, Andreetti C, Tierno S, et al. Margin distance does not influence recurrence and survival after wedge resection for lung cancer. In: Annals of Thoracic Surgery [Internet]. Elsevier USA; 2015 [cited 2021 Mar 19]. p. 918–25. Available from: https://pubmed.ncbi.nlm.nih.gov/26209486/

13. Black WC, Gareen IF, Soneji SS, Sicks JD, Keeler EB, Aberle DR, et al. Cost-Effectiveness of CT Screening in the National Lung Screening Trial. N Engl J Med [Internet]. 2014 Nov 6 [cited 2019 Dec 11];371(19):1793–802. Available from: http://www.nejm.org/doi/10.1056/NEJMoa1312547
